# Mitochondrial Whole D-Loop Variability in Polish Draft Horses of Sztumski Subtype

**DOI:** 10.3390/ani12151870

**Published:** 2022-07-22

**Authors:** Grzegorz Myćka, Weronika Klecel, Monika Stefaniuk-Szmukier, Joanna Jaworska, Adrianna Dominika Musiał, Katarzyna Ropka-Molik

**Affiliations:** 1 Department of Biotechnology and Horticulture, University of Agriculture in Kraków, al. 29 Listopada 54, 31-425 Kraków, Poland; grzegorz.mycka@student.urk.edu.pl; 2 Department of Animal Genetics and Conservation, Institute of Animal Science, Warsaw University of Life Sciences, ul. Ciszewskiego 8, 02-786 Warsaw, Poland; weronika_klecel@sggw.edu.pl; 3 Department of Animal Genomics and Molecular Biology, National Research Institute of Animal Production, ul. Krakowska 1, 32-083 Balice, Poland; adrianna.musial@iz.edu.pl (A.D.M.); katarzyna.ropka@iz.edu.pl (K.R.-M.); 4 Department of Gamete and Embryo Biology, Institute of Animal Reproduction and Food Research, Polish Academy of Sciences, 10-747 Olsztyn, Poland; j.jaworska@pan.olsztyn.pl

**Keywords:** mtDNA, whole d-loop, Polish cold-blood horse, equine

## Abstract

**Simple Summary:**

The Polish draft horse (PDH) breed is a result of crossing local mares with imported cold-blooded stallions. In this study, we investigate the genetic composition of the PDH by analyzing whole mitochondrial d-loop variabilities and comparing these variabilities to previously demonstrated whole d-loop sequences of other cold-blooded breeds: Ardennais, Belgian, Breton, Clydesdale, Noriker, Norwegian Fjord, Percheron, and Suffolk. Our results show high nucleotide diversity among the PDH population and the existence of two main haplogroups with strong kinship to the Belgian breed.

**Abstract:**

The Polish draft horse (PDH) breed is a result of crossing local mares with imported cold-blooded stallions, such as Belgians, Ardennes, Fjords, and others. A part of the broodmare stock investigated in this study was also imported from various countries, such as Denmark. In this study, we investigate the genetic composition of the PDH by analyzing the whole mitochondrial d-loop variability and comparing it to previously demonstrated whole d-loop sequences of other cold-blooded breeds: Ardennais, Belgian, Breton, Clydesdale, Noriker, Norwegian Fjord, Percheron, and Suffolk. Our results show high nucleotide diversity within the PDH population (π = 0.011), and the existence of two main haplogroups: one of relatively concise origin, with strong kinship to the Belgian breed, and the second showing close relation to the majority of other analyzed cold-blooded breeds. Some of the PDH maternal strains clustered separately, which can be a result of the influence of other unidentified breeds that served as a foundation stock for the present population. This present study explains the genetic relationship of the PDH to other cold-blooded breeds and indicates the high genetic diversity of the breed.

## 1. Introduction

Draft horses (heavy horses, cold-blooded horses, etc.) are undoubtedly part of European culture. For centuries, the horses’ phenotypes have been influenced by breeder selection to meet the requirements of the tasks the animals were being bred to perform. Until the Industrial Revolution, horses were used for transportation, the military, agriculture, and heavy industry purposes. However, with the progress of mechanization, especially in the areas of military, agriculture, and heavy industry, followed by the World Wars, the number of horses globally has dramatically decreased. Their selection goals have shifted towards leisure and sport [1]. The populations of heavy working horse breeds are limited, and some of them have disappeared forever (f. ex. Łużycki in Poland). Plenty of international efforts have been made to maintain several populations under the FAO (Food and Agriculture Organization of the United Nations) to preserve the gene pool and to protect the horses’ biodiversity and cultural heritage; however, these actions were conservative and had no relative impact on the self-reconstruction of populations [2].

The Polish cold-blooded horse subpopulation began to form in the middle of the 19th century based on native, working horses, refined with imported cold-blooded horses, mainly from Belgium, France, and Germany [3]. The name “cold-blooded horse” appeared in Polish professional literature in 1964 with the publication of the first Stud Book. Conservation programs are being implemented to preserve this breed. According to a statistic published by the Polish Horse Association in 2020, the reproduction stock was roughly 12 thousand animals (“Breeding statistic. Available online: https://www.pzhk.pl/hodowla/statystyka-hodowlana/ (accessed on 5 May 2022)”). However, the organization of animal production in the period between 1945 and 1989 focused mainly on State Studs, with the private breeding sector maintaining horses for work with little addition for breeding. As systematic breeding work was carried out, there was a genetic consolidation of the herd and phenotypic equalization of the population. Recently, that breed emerged into two subtypes, named Sokólski and Sztumski [4], which are currently under a breeding conservation program. The modern breeding conservation programs derive information from genetic data describing levels of diversity, as well as origin influence of other breeds and, thus, are the tools for the effective management of populations [5]. Presently, the Polish draft horse studbook allows the conditional entries of Ardennes, Belgian, German cold-blood, Percheron, Breton, Norwegian Fjord, and Slovenian cold-blood [3].

Some recently evolved tools in the field of molecular genetics have led to the intensive study of the genetic diversity of breeds, which is crucial for maintaining the endangered and low-numbered breeds [6]. However, one of the earliest-explored, most useful, and most informative tools is investigating the mitochondrial DNA diversity in population genetics and molecular phylogenetics. Since it is maternally inherited, haploid, non-recombining, and possesses an elevated mutation rate compared to nuclear DNA, it has been used to determine intra- and inter-genetic variation within various breeds and species. Furthermore, mitochondrial DNA is extensively used to genetically distinguish one dam line from another and to trace ancestry through the maternal lineage of a pedigree to verify the reliability of the maternal lineages recorded in the Studbooks using mitochondrial diversity analysis [7,8,9].

The mtDNA in equines is a 16,660 bp circular genome, containing 13 intron-less protein-coding genes that are part of 5 protein complexes, 22 transfer RNAs, 2 ribosomal RNAs, and the control region (d-loop, displacement region). The d-loop itself is approx. 1200 bp long, containing the regulatory elements for replication and transcription with two hypervariable segments (HVR1 and HVR2), a large conserved sequence block, and three small conserved sequence blocks with tandem repeats of an 8 bp equine-specific sequence TGTGCACC between [10,11]. This part of the genome is extensively studied in the research on maternal lineages.

To date, most studies focused on the most variable region of mtDNA, the approximately 350–600 bp-long hypervariable region 1 (HVR1) of the d-loop. This part has been sequenced to explore the diversity of different breeds from past and present, including Italian Heavy Draught, Sardinian Anglo-Arab, Finnish, Tibetan, Polish Konik, and many others horse breeds [7,12,13,14,15]. Among them, only a few investigated the maternal origin of coldblooded horses. Moreover, all of these studies used only a limited part of the d-loop and dealt with only a small number of phylogenetically informative sites. The previous study investigating the genetic structure and phylogenetic relationships of the Polish draft horse focused on a small 421 bp fragment of the d-loop region, and no phylogenetic patterns were detected [16]. Therefore, the main purpose of the present study is to identify the maternal origin of modern Polish draft horses of Sztumski subtype, based on the whole d-loop sequences. 

## 2. Materials and Methods

### 2.1. Ethics Statement

The hair follicles (between 5 and 10 per sample) or whole blood samples from all horses came from the same collections of the reference horse database of the Laboratory of Molecular Genetics, Department of Animal Molecular Biology, and the Biological Material Bank of the National Research Institute of Animal Production. The experimental protocol was approved by the Animal Care and Use Committee of the Institute of Pharmacology, Polish Academy of Sciences in Cracow (no. 1173/2015).

### 2.2. Animals 

A total of 72 horses were included in the study. All horses were entered into the main section of the Polish cold-blood Horse Studbook and were the property of a strategic state-owned company, specializing in PDH (Sztumski type) breeding. The sample consisted of all horses available at the farm at the moment of the sample collection. The biological material collected for the purposes of the study was 5 ml of jugular blood, collected to sterile tubes with EDTA as an anticoagulant, or hair follicle. 

To identify more complex phylogenetic relationships, we additionally retrieved all the complete d-loop sequences available in GenBank (*n* = 26), including breeds indicated as ancestral or related to Polish cold-bloods (Belgian (*n* = 9), Norwegian Fjord (*n* = 2), Breton (*n* = 1), Percheron (n = 1), Ardennais (*n* = 1), and Suffolk (*n* = 1)) as well as other modern cold-blood breeds (Noriker (*n* = 5) and Clydesdale (*n* = 6)). The GenBank X79547.1 *Equus caballus* reference sequence [10] was retrieved as an outgroup for phylogenetic analysis. A list of all breeds and sequence accession numbers is provided in Table S1 [17,18,19,20,21].

### 2.3. DNA Extraction and Sanger Sequencing

Genomic DNA was purified using a Sherlock AX kit (A&A Biotechnology, Gdańsk, Poland) according to the manufacturer’s protocol, with the use of 100 µL of whole blood or hair follicle. The quality and quantity of extracted DNA were measured with a NanoDrop 2000 spectrophotometer (Thermofisher Scientific, Waltham, MA, USA). For the detection of variation, primers covering the hypervariable regions (HVR1-HVR2) of the d-loop were designed using the Primer3Plus program (“Software. Available online: www.primer3plus.com (accessed on 5 July 2021)”) [22], with the GenBank X79547.1 sequence as a reference. The detailed primer sequences are provided in Table 1. Furthermore, primers were designed to avoid the amplification of nuclear mitochondrial DNA sequences (NUMTs) [23] and to cover two HVR regions. The HVR1A and HVR1B primers cover 716 bp of HVR1 and 263 bp of HVR2, given 979 bp of 1200 whole d-loop sequence. The unsequenced non-hypervariable region (16137 – 16357) was filled complementarily to the reference sequence. The PCR reaction was performed with an AmpliTaq Gold 360 Master Mix (Life Technologies, Carlsbad, CA, USA), at an annealing temperature of 56 °C. The obtained PCR product was purified of free nucleotides and primers using EPPiC (A&A Biotechnology) and then prepared for sequencing using the BigDye™ Terminator v3.1 Cycle Sequencing Kit (Life Technologies California, Carlsbad, CA, USA). Then, the products were purified using the BigDye XTerminator Purification Kit (ThermoFisher Scientific). Sequencing was performed using a Genetic Analyzer 3500x1 capillary sequencer (Applied Biosystems, Waltham, MA, USA) according to the protocol. To check the quality of the sequencing, the sequences were compared using the BLAST algorithm “Software. Available online: https://blast.ncbi.nlm.nih.gov, (accessed on 13 September 2021)” [24]. The sequences were read using FinchTV 1.3.0 (Geospiza, Inc.; Seattle, WA, USA 2004–2005) and compared with a reference sequence (GenBank X79547.1). The unsequenced non-hypervariable region (16137–16357) was completed complementarily to the reference sequence.

### 2.4. Statistical Analysis 

The numbers of segregating and parsimony-informative sites, as well as haplotype diversity and nucleotide diversity (intra- and inter-populations), were calculated using DnaSP v.6 [25]. The neutral mutation hypothesis was tested with Tajima’s D test.

To visualize the phylogenetic relationship between analyzed haplotypes, we constructed the neighbor-joining tree using Mega11 [26], as well as the median-joining network [27] (with tolerance parameter ε = 0) using PopART software [28]. The bootstrap consensus tree inferred from 1000 replicates was taken to represent the phylogenetic relationship between the Polish draft horse and related cold-blooded breeds. Branches corresponding to partitions reproduced in less than 50% of bootstrap replicates were collapsed. The percentage of replicate trees in which the associated taxa clustered together in the bootstrap test (1000 replicates) is shown next to the branches. The distances between analyzed haplotypes were computed using the p-distance method and presented in the units of the number of base differences per site. The analysis involved 41 nucleotide sequences: 15 new sequences of Polish draft horse and 26 complete d-loop sequences obtained from the GenBank database. Nineteen positions containing gaps and missing data were eliminated (complete deletion option). A total of 957 positions were in the final dataset.

## 3. Results

The raw sequence obtained by Sanger Sequencing and sequences from GenBank were trimmed for comparison, and finally, the part of mitochondrial DNA located between 15490 and 16650 bp (1160 sites) of the entire loop was included for further analysis. The novel sequences of investigated maternal lines were deposited in GenBank under the numbers: ON052711–ON052725.

Among the segregating sites, we also identified four mutational hotspots previously described located at positions 15585, 15597, 15650, and 15659 [29], and these positions were not included in phylogenetic analysis (Table 2A,B).

Based on the sequencing results, Polish draft horses included in this study clustered into 15 distinct haplotypes (HapDiv = 0.933; sd = 0.081).

The variation analysis of the Polish draft horse subpopulation revealed 46 segregating sites and 32 parsimony-informative sites, which corresponds with Tajima’s D = −0.114 (*p* = 0.940). The nucleotide diversity reached the value of π = 0.011.

**Figure 1:**
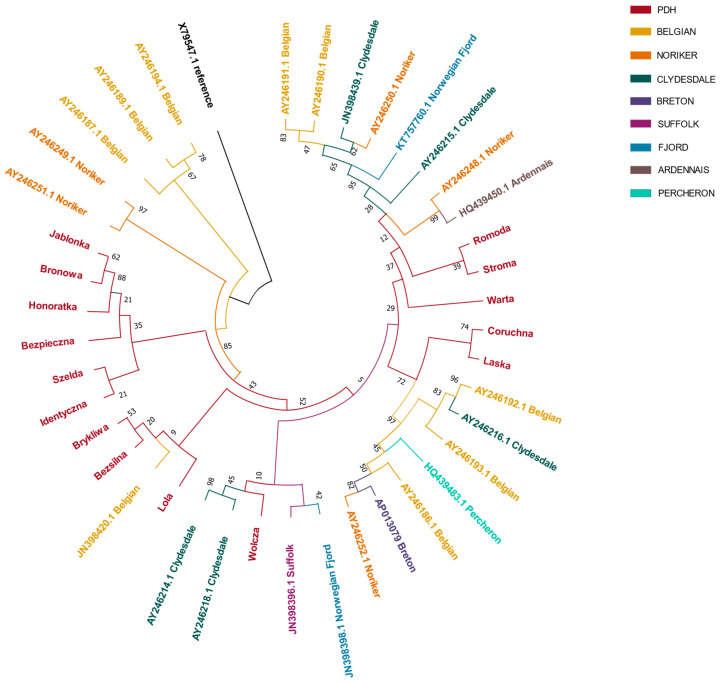
A neighbor-joining tree for Polish Draft Horses and related cold-blooded breeds. The GenBank X79547.1 reference sequence is used as a root of the tree. Each breed is represented by a different color.

The consensus neighbor-joining tree (Figure 1) was constructed based on the analysis of 41 nucleotide sequences. The GenBank X79547.1 reference sequence was used as a root of a tree. Fifteen new haplotypes of Polish draft horse were compared to 26 complete d-loop sequences obtained from the GenBank database. The neighbor-joining tree clearly showed the complex, multi-breed origin of Polish draft horse matrilines. While six of the haplotypes clustered in a separate clade, the remaining nine showed phylogenetic similarity to other breeds included in the analysis: close proximity to Belgian, Clydesdale, Suffolk, and Norwegian Fjord, as well as sharing a common ancestor with Percheron, Breton, Ardennais, and Noriker breeds.

**Figure 2:**
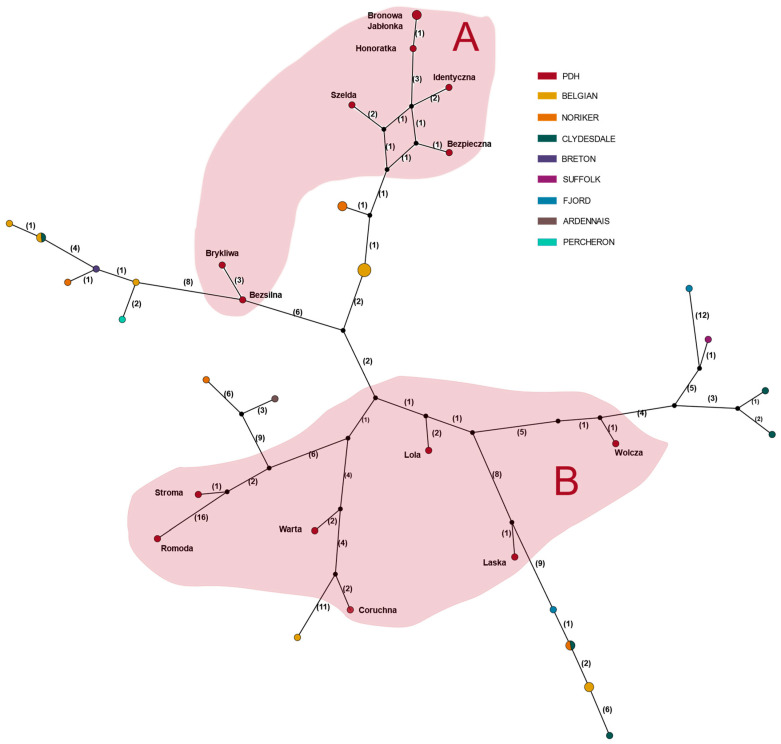
A median-joining network of the modern breeds haplotypes (*n* = 41). Each breed is represented by a different color. Haplotypes that are separated by only one segregating side are clustered together. The size of the circles represents the number of haplotypes that clustered together. Sizes of the nodes are proportional to the numbers of parsimony-informative sites defining the haplotypes, which are given in the numbers in parentheses. Haplogroups of Polish draft horse are marked with the corresponding color.

Furthermore, the median-joining network (Figure 2) demonstrates the genetic distance between analyzed haplotypes regarding the number of polymorphic sites. Based on the figures, we were able to identify two main haplogroups of Polish draft horse. Group A shows the largest homogeneity, as well as propinquity to the uniform Belgian horse haplogroup. Group B may be characterized as multi-origin, with high genetic diversity.

## 4. Discussion

The current population of the Polish draft horse developed from local mares crossed with stallions mostly delivered to Poland by the UNRRA (United Nations Relief and Rehabilitation Administration) and imported from Belgium, Denmark, Finland, the Netherlands, Norway, and Sweden [16]. Consistent breeding work has originated a consolidated type of cold-blooded horse. However, the final shape of the breed is influenced by various environmental factors, namely the type of usage, whether economic, social, political, or cultural. These factors influenced the production of many local types of horses. Presently, only two types of Polish draft horse, namely Sokólski and Sztumski, remain actively breeding, and they are both under the National Program for Protection of Genetic Resources [30]. Since 1964, when the first volume of the PDH studbook was published, two separate sections of the studbook have been maintained in Poland due to a clear heritable difference in type and phenotypical traits and genetic differentiation [2]. In this study, the analysis of whole d-loop variation of mtDNA was applied to investigate the origin and variability of the PDH maternal lines that have been formed over multiple generations.

Among all the analyzed Polish cold-blood horses, 15 distinct haplotypes have been determined with quite a high percentage of diversity. Bronowa and Jabłonka were separated by only one segregating site, so the difference between these two haplotypes may be a result of a genetic drift [31]. Overall, two main haplogroups were visible, of which one was highly diverse. These splits are evidence of the complex origin of the cold-blooded horse as a group of breeds. Considering connections with contemporary cold-blooded breeds, the closest and most straightforward relationship is found with the Belgians—the oldest currently known cold-blooded breed. Equally essential bindings were visible with the Bretons and Fjords—both breeds being present-day cold-blooded horses from northern Europe. All of these breeds were previously indicated as ancestral to the PDH [6]. While relatively distant in terms of nucleotide composition, damline haplotypes for Laska, Curóchna, and Warta show common ancestry, which provides evidence of significant external influences in previous generations. Haplotypes for lines Szelda and Bezpieczna clustered closely with the Norikers—a mid-European breed, originally from the Roman Empire, and very popular in Austria and German Bavaria throughout the 19th century [32].

Understanding the mode of inheritance of mtDNA through only maternal lines leads to an extended image of the cold-blooded horse genetic pool. The group, consisting of many breeds representing different origins, has been interbred for centuries. After the analysis of numerous genomes, the relationships between them become visible, as well as the role of a single group in the whole breed phylogeny. We believe that including the mtDNA information in the studbook would benefit the breeding management of any horse breed, including the PDH.

Determining more detailed phylogenetic history of the modern PDH may be an interesting subject for further research, as some of the analyzed haplotypes clustered in separate branches of the phylogenetic tree. However, sequences of the potential maternal ancestors representing local, more primitive types may be difficult to obtain, as the majority of Polish primitive horses became extinct more than 50 years ago.

## 5. Conclusions

Our results have shown the uniqueness of the Polish draft horses’ mitochondrial genome among other breeds and confirmed the multi-breed admixture in the gene pool. Poland’s geographical location in the center of the continent gave the populations a great opportunity for genetic exchange in multiple directions. Polish draft horses used in the present study may be considered relicts with their rich genealogy and, thus, deserve special protection in terms of genetic resources.

## Figures and Tables

**Table 1:** Primer sequences used in the study: hypervariable region 1 (divided in two parts: A and B), and hypervariable region 2, with its product lengths.

Primer	Region Amplified	Sequence	Product Length (bp)
HVR1A	15382–15778	F: AACGTTTCCTCCCAAGGACT R: GTAGTTGGGAGGGTTGCTGA	397
HVR1B	15676–16137	F: ACCCCATCCAAGTCAAATCA R: CAGGTGCACTTGTTTCCTATG	461
HVR2	16357–16660	F: ACCTACCCGCGCAGTAAGCAA R: ACGGGGGAAGAAGGGTTGACA	304

**Table 2:** A: All Polish draft horse haplotype sequences used in the study with the Single Nucleotide Polymorphisms (SNPs) between positions 15490 and 15766, compared with the reference sequence X79547.1. *—hotspots; ⸱—no differences. B: All Polish draft horse haplotype sequences used in the study with the Single Nucleotide Polymorphisms (SNPs) between positions 15769 and 16629, compared with the reference sequence X79547.1; ⸱—no differences.

**A**	**Damline Name**	**Haplotype (Achilli et al. 2012 [19])**	**n**	**15490**	**15494**	**15495**	**15496**	**15534**	**15538**	**15542**	15585 *	15597 *	**15602**	**15604**	**15617**	**15635**	**15649**	15650 *	15659 *	**15666**	**15667**	**15700**	**15703**	**15709**	**15720**	**15762**	**15766**
				**C**	**T**	**T**	**A**	**C**	**A**	**C**	**G**	**A**	**C**	**G**	**T**	**C**	**A**	**A**	**T**	**G**	**A**	**C**	**T**	**C**	**G**	**G**	**C**
Haplotypes	Bezpieczna	A	4	⸱	⸱	⸱	⸱	⸱	⸱	⸱	⸱	⸱	⸱	⸱	⸱	⸱	⸱	⸱	⸱	⸱	⸱	⸱	⸱	⸱	⸱	⸱	⸱
	Bezsilna	G	2	⸱	⸱	⸱	⸱	⸱	⸱	T	⸱	G	T	⸱	⸱	T	⸱	⸱	C	⸱	⸱	⸱	⸱	⸱	⸱	⸱	⸱
	Bronowa	L	2	⸱	⸱	C	⸱	⸱	⸱	⸱	A	⸱	⸱	⸱	⸱	⸱	⸱	G	⸱	A	⸱	⸱	⸱	⸱	A	⸱	⸱
	Brykliwa	A	2	⸱	⸱	C	⸱	⸱	⸱	T	⸱	G	T	⸱	⸱	T	⸱	G	⸱	A	⸱	⸱	C	⸱	A	⸱	⸱
	Córuchna	L	2	⸱	⸱	⸱	G	T	⸱	⸱	A	⸱	⸱	A	⸱	⸱	G	⸱	⸱	⸱	⸱	⸱	⸱	⸱	⸱	⸱	⸱
	Honoratka	A	2	⸱	⸱	C	⸱	⸱	⸱	⸱	A	⸱	⸱	⸱	⸱	⸱	⸱	G	⸱	A	⸱	⸱	⸱	⸱	A	⸱	⸱
	Identyczna	A	14	T	⸱	⸱	⸱	⸱	⸱	⸱	⸱	⸱	⸱	⸱	⸱	⸱	⸱	⸱	⸱	⸱	⸱	⸱	⸱	⸱	⸱	⸱	⸱
	Jabłonka	A	7	⸱	⸱	C	⸱	⸱	⸱	⸱	A	⸱	⸱	⸱	⸱	⸱	⸱	G	⸱	A	⸱	⸱		⸱	A	⸱	⸱
	Lola	B	10	⸱	⸱	⸱	⸱	⸱	⸱	⸱	⸱	⸱	⸱	⸱	⸱	⸱	⸱	⸱	⸱	⸱	⸱	⸱	⸱	⸱	⸱	⸱	⸱
	Łaska	G	12	⸱	C	⸱	G	⸱	⸱	⸱	⸱	⸱	⸱	⸱	⸱	⸱	⸱	⸱	⸱	⸱	⸱	⸱	⸱	⸱	⸱	⸱	⸱
	Romoda	B	2	⸱	⸱	C	⸱	⸱	⸱	⸱	A	⸱	T	A	⸱	⸱	⸱	⸱	⸱	⸱	G	⸱	C	⸱	A	⸱	⸱
	Stroma	B	3	⸱	⸱	C	⸱	⸱	⸱	⸱	A	⸱	T	⸱	C	⸱	⸱	⸱	C	⸱	⸱	T	C	⸱	A	A	T
	Szelda	P	6	⸱	⸱	⸱	⸱	⸱	G	⸱	⸱	⸱	⸱	⸱	⸱	⸱	⸱	G	⸱	⸱	⸱	⸱	⸱	⸱	⸱	⸱	⸱
	Warta	M	2	⸱	⸱	⸱	⸱	⸱	⸱	⸱	⸱	⸱	⸱	⸱	C	⸱	⸱	⸱	C	⸱	⸱	⸱	⸱	⸱	⸱	⸱	⸱
	Wołcza	I	2	⸱	⸱	C	⸱	⸱	G	⸱	A	⸱	T	⸱	⸱	⸱	⸱	⸱	⸱	⸱	⸱	⸱	⸱	T	A	⸱	⸱
**B**	**Reference X79547.1**	**Haplotype (Achilli et al. 2012)**	**n**	**15769**	**15770**	**15771**	**15801**	**15807**	**15810**	**15811**	**15826**	**15827**	**15870**	**15871**	**15956**	**15974**	**15995**	**16007**	**16022**	**16031**	**16055**	**16071**	**16543**	**16546**	**16558**	**16559**	**16629**
				**T**	**C**	**C**	**C**	**C**	**A**	**C**	**A**	**A**	**C**	**C**	**A**	**C**	**A**	**T**	**T**	**T**	**A**	**T**	**T**	**T**	**ins**	**C**	**A**
Haplotypes	Bezpieczna	A	4	⸱	⸱	⸱	⸱	⸱	G	⸱	G	⸱	⸱	⸱	⸱	⸱	⸱	⸱	⸱	⸱	G	⸱	⸱	⸱	⸱	⸱	⸱
	Bezsilna	G	2	⸱	⸱	⸱	⸱	⸱	⸱	⸱	⸱	⸱	T	⸱	⸱	⸱	⸱	⸱	⸱	C	⸱	⸱	⸱	⸱	C	⸱	⸱
	Bronowa	L	2	⸱	⸱	⸱	⸱	⸱	G	⸱	G	⸱	⸱	⸱	⸱	⸱	⸱	⸱	⸱	⸱	G	⸱	⸱	⸱	⸱	⸱	⸱
	Brykliwa	A	2	⸱	⸱	T	⸱	⸱	⸱	⸱	⸱	G	T	T	⸱	⸱	⸱	⸱	⸱	⸱	⸱	⸱	⸱	⸱	⸱	⸱	⸱
	Córuchna	L	2	⸱	⸱	⸱	⸱	⸱	⸱	⸱	⸱	⸱	⸱	⸱	G	⸱	⸱	⸱	⸱	⸱	⸱	⸱	⸱	⸱	C	⸱	G
	Honoratka	A	2	⸱	⸱	⸱	⸱	⸱	⸱	⸱	G	⸱	⸱	⸱	⸱	⸱	⸱	⸱	⸱	⸱	G	⸱	⸱	⸱	⸱	⸱	⸱
	Identyczna	A	14	⸱	⸱	⸱	⸱	T	⸱	⸱	⸱	⸱	⸱	⸱	⸱	⸱	⸱	⸱	⸱	⸱	⸱	⸱	⸱	⸱	C	⸱	⸱
	Jabłonka	A	7	⸱	⸱	⸱	⸱	⸱	G	⸱	G	⸱	⸱	⸱	⸱	⸱	⸱	⸱	⸱	⸱	G	⸱	⸱	⸱	⸱	⸱	⸱
	Lola	B	10	⸱	⸱	T	T	⸱	⸱	⸱	⸱	G	⸱	⸱	⸱	⸱	⸱	⸱	⸱	⸱	⸱	C	⸱	⸱	⸱	⸱	⸱
	Łaska	G	12	⸱	⸱	⸱	⸱	⸱	⸱	⸱	⸱	⸱	⸱	⸱	G	⸱	⸱	⸱	⸱	⸱	⸱	⸱	⸱	⸱	C	⸱	G
	Romoda	B	2	⸱	T	T	⸱	⸱	⸱	⸱	⸱	⸱	T	T	G	⸱	⸱	⸱	⸱	⸱	⸱	⸱	A	⸱	C	⸱	G
	Stroma	B	3	C	T	⸱	⸱	T	⸱	T	⸱	G	T	T	⸱	⸱	G	C	C	⸱	⸱	⸱	A	C	⸱	T	G
	Szelda	P	6	⸱	⸱	⸱	⸱	⸱	⸱	⸱	⸱	⸱	⸱	⸱	⸱	⸱	⸱	⸱	⸱	⸱	G	⸱	⸱	⸱	⸱	⸱	⸱
	Warta	M	2	⸱	⸱	⸱	⸱	⸱	⸱	⸱	⸱	⸱	⸱	⸱	⸱	⸱	⸱	⸱	⸱	⸱	⸱	⸱	A	C	⸱	T	G
	Wołcza	I	2	⸱	⸱	T	⸱	⸱	⸱	⸱	G	G	T	T	G	T	⸱	⸱	⸱	⸱	⸱	⸱	⸱	⸱	C	⸱	⸱

## Data Availability

Raw data are available upon request from the corresponding authors.

## Supplementary Materials

The following supporting information can be downloaded at: https://www.mdpi.com/article/10.3390/ani12151870/s1, Table S1: Identification numbers of the sequences retrieved from GenBank for the purpose of this study.
