# Supplementary material for: Mitochondrial Whole D-Loop Variability in Polish Draft Horses of Sztumski Subtype
Source: Animals (Basel). 2022 Jul 22;12(15):1870. doi: 10.3390/ani12151870 (PMC9332387; doi:10.3390/ani12151870)
Supplement: Supplementary file 1 [file animals-12-01870-s001.zip › animals-1779058-supplementary.pdf]

Table S1: Identification numbers of the sequences retrieved from GenBank for the purpose of this study.

Breed	Sequence number(s)	Source
Ardennais	HQ439450	[17]
Belgian	AY246186, AY246187, AY246189-AY246194	[18]
	JN398420	[19]
Breton	AP013079	[20]
Clydesdale	AY246214, AY246215, AY246218	[18]
	JN398439	[19]
Fjord	JN398398	[19]
	KT757760	[21]
Noriker	AY246248-AY246252	[18]
Percheron	HQ439483	[18]
Suffolk	JN398396	[20]
